# Intramuscular Myxoma of the Intrinsic Muscles of the Tongue: A Case Report with Literature Review

**DOI:** 10.1155/2022/7067949

**Published:** 2022-10-12

**Authors:** Naoko Tsunoda, Kei Onodera, Yu Ohashi, Tadashi Kawai, Ikuya Miyamoto, Yasunori Takeda, Hiroyuki Yamada

**Affiliations:** ^1^Division of Oral and Maxillofacial Surgery, Department of Oral and Macillofacial Reconstructive Surgery, School of Dentistry, Iwate Medical University, Morioka 020-8505, Japan; ^2^Division of Clinical Pathology, Department of Oral and Maxillofacial Reconstructive Surgery, School of Dentistry, Iwate Medical University, Morioka 020-8505, Japan

## Abstract

Myxoma is a benign tumor of mesenchymal origin. It frequently occurs in the muscles of the hip and extremities; however, it rarely occurs in the head and neck region. This report describes the second case of an intramuscular myxoma of the tongue. A 23-year-old woman was referred to our institution for the diagnosis and treatment of a left tongue lesion. T2-weighted magnetic resonance imaging revealed an 8 × 6-mm mass in the tongue. Based on a clinical diagnosis of a tongue tumor, excisional biopsy was performed under general anesthesia. The histopathological diagnosis was an intramuscular myxoma. The postoperative course was uneventful, and there was no evidence of tumor recurrence 3 years after surgery.

## 1. Introduction

Myxoma is a benign tumor of mesenchymal origin. It frequently occurs in the muscles of the hip and extremities; however, it rarely occurs in the head and neck region [1]. The symptoms of myxoma include indolent swelling and a slowly growing mass. Myxomas show a female predilection, and most cases are diagnosed between 40 and 70 years of age [1]. No reports have described malignant transformation or metastasis of a myxoma.

In the head and neck region, odontogenic myxomas develop in the maxilla or mandible. They are neoplasms of odontogenic origin and thus differ from soft tissue myxoma, which is classified as a tumor of uncertain differentiation [2]. Because of the rarity of soft tissue myxomas, most clinicians are unfamiliar with their clinic findings.

We herein report the second case of an intramuscular myxoma of the tongue, together with a literature review.

## 2. Case Presentation

**Figure 1:**
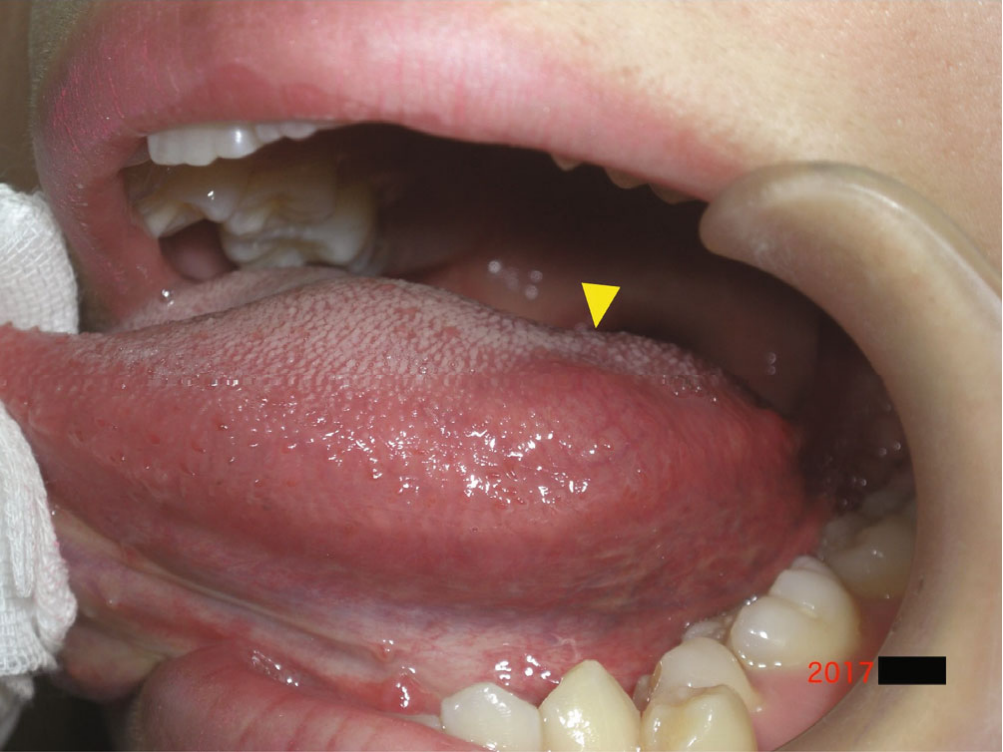
Intraoral photograph showing an elastic soft mass measuring 10 mm in diameter in the left tongue (arrowhead). The overlying mucosa is normal.

A 23-year-old woman was referred to our institution for the diagnosis and treatment of a left tongue lesion. She had first noticed the lesion approximately 1 month earlier. The patient's medical and family histories were unremarkable. Intraoral examination revealed a 10-mm-diameter elastic soft mass in the left tongue. The overlying mucosa was normal (Figure 1). There was no evidence of cervical lymphadenopathy.

**Figure 2:**
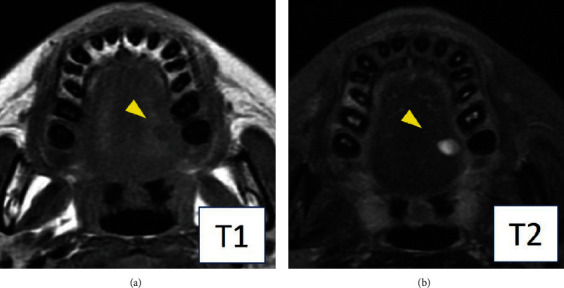
Magnetic resonance imaging findings. (a) T1-weighted axial image showing low signal intensity within the lesion (arrowhead). (b) T2-weighted axial image showing increased signal intensity within the lesion (arrowhead).

Magnetic resonance imaging (MRI) was performed with a 3.0-Tesla system (MR750; General Electric Company, Boston, MA, USA). On T1-weighted axial images, the mass in the left tongue showed low signal intensity relative to muscle. On T2-weighted images, increased signal intensity was noted within the lesion (Figure 2). The lesion showed a homogeneous appearance. The size of the mass was 8 × 6 mm. No tumorous lesion was obvious in either the maxilla or mandible.

**Figure 3:**
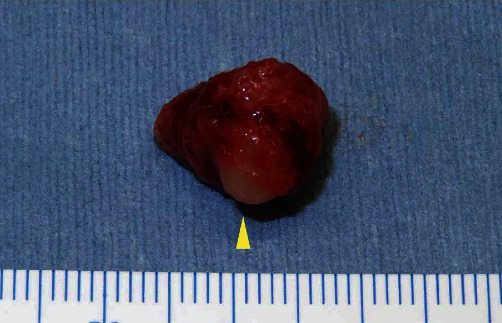
Photograph showing the resected tumor (arrowhead) surrounded by the muscle of the tongue.

**Figure 4:**
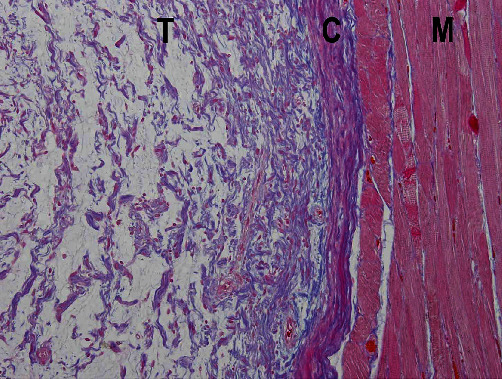
Photomicrograph showing stellate and spindle-shaped cells with no atypia in the abundant myxoid extracellular matrix (hematoxylin–eosin stain, ×100). T: tumor, C: fibrous capsule, M: muscle of the tongue.

**Figure 5:**
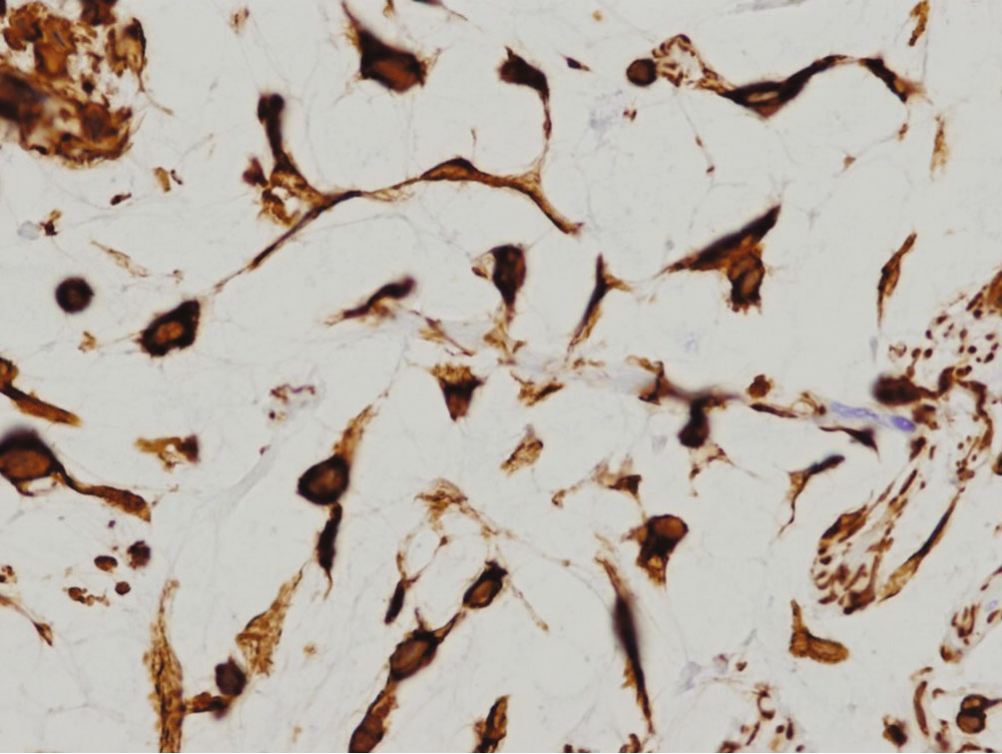
Histopathologic photograph showing the results of immunohistochemical analyses for vimentin, which is positive in the tumor cells (vimentin, ×400).

Based on a clinical diagnosis of a benign tongue tumor, excisional biopsy was performed under general anesthesia. A spindle-shaped incision was made on the lateral surface of the left tongue. The specimen was pulled outward with slight lateral traction and cut using a surgical scalpel. Finally, the whole tumor with surrounding soft tissue was obtained as a surgical specimen (Figure 3). Histopathological examination of the resected tumor revealed that the tumor within the muscle was encapsulated by fibrous connective tissue. Stellate and spindle-shaped cells with no atypia were observed within an abundant myxoid extracellular matrix (Figure 4). No mitotic figures were present. Immunohistochemical analysis revealed that the tumor cells were positive for vimentin (Figure 5) and negative for desmin and cyclin-dependent kinase 4. Thus, the histopathological diagnosis was an intramuscular myxoma.

The postoperative course was uneventful. There was no evidence of tumor recurrence 3 years after surgery.

## 3. Discussion

Intramuscular myxomas in the head and neck are rare. To the best of our knowledge, 33 cases of intramuscular myxomas in the head and neck, including the present case, have been reported in the literature to date [3–34]. These cases are summarized in Table 1. The patients comprised 14 men and 19 women, and their mean age was 49.0 years (range, 2–86 years). The most frequently involved site was the posterior neck in nine patients, followed by the masseter muscle in four patients. Most cases were sporadic; however, one patient had multiple lesions [10]. The maximum length of the tumor ranged from 0.6 to 15.0 cm, and the tumor in the present case was the second smallest.

Typical MRI findings of intramuscular myxomas are homogeneous low signal intensity in T1-weighted images and high signal intensity in T2-weighted images [35]. Luna et al. [36] reported that 7 of 18 intramuscular myxomas showed an inhomogeneous appearance in T2-weighted images, probably because fibrous internal septa are sometimes seen in intramuscular myxomas. MRI was performed in 12 of 33 intramuscular myxomas in the head and neck region. Nine of these 12 cases showed low to intermediate signal intensity in T1-weighted images and high signal intensity in T2-weighted images [20, 22, 24, 26, 27, 29, 31, 33]. In addition, internal septa were recognized in two large tumors [20, 22]. However, these findings are nonspecific; therefore, no cases were diagnosed as an intramuscular myxoma on MRI.

Preoperative fine needle aspiration cytology was performed in 8 of 33 intramuscular myxomas in the head and neck. Only one case was correctly diagnosed as a myxoma [13]. A diagnosis of myxoma was not reached in the remaining seven cases [12, 15, 18, 20, 23, 26, 34]. Therefore, it is difficult to achieve the correct diagnosis of myxoma by fine needle aspiration cytology.

The histopathological differential diagnoses of intramuscular myxoma include low-grade myxofibrosarcoma, myxoid liposarcoma, nerve sheath myxoma, and oral focal mucinosis. Low-grade myxofibrosarcoma exhibits infiltrative behavior [2]; however, the tumor in the present case was encapsulated by fibrous connective tissue. Although myxoid liposarcoma contains a prominent myxoid stroma, its characteristic findings of moderate cellularity and lobulated morphology are not seen in intramuscular myxoma [2]. Nerve sheath myxoma is a benign peripheral nerve sheath tumor composed of small epithelioid, ring-like, and spindled Schwann cells [2]. The findings of a multinodular growth pattern and epithelioid Schwann cells arranged in cords can distinguish nerve sheath myxoma from intramuscular myxoma. Oral focal mucinosis is non-neoplastic lesion that likely develops secondary to overproduction of hyaluronic acid by fibroblasts [37]. Fragmented and randomly arranged ropey collagen fibers seen in oral focal mucinosis can help to differentiate it from intramuscular myxoma. In the present case, the encapsulated tumor in the muscle was composed of stellate and spindle-shaped cells with no atypia within an abundant myxoid extracellular matrix. In addition, the tumor cells were negative for desmin and cyclin-dependent kinase 4. These findings were consistent with the diagnosis of intramuscular myxoma.

Mazabraud syndrome, which was first reported by the French physician Mazabraud [38] in 1967, is characterized by the combination of multiple intramuscular myxomas and fibrous dysplasia. Care should be taken to avoid overlooking this lesion because Fu et al. [39] reported a sporadic intramuscular myxoma of the masseter muscle with mandibular fibrous dysplasia. In the present case, the MRI findings ruled out fibrous dysplasia in the head and neck; however, other bone sites were not screened because of the absence of clinical symptoms. Therefore, the possibility of Mazabraud syndrome was not completely excluded in the present case.

An intramuscular myxoma in the head and neck region is treated by surgical excision. Recurrence is rare; only two myxomas in the posterior neck [9] and in the lip [14] recurred after surgery. In the case of the posterior neck myxoma [9], the tumor recurred 2 months after the operation because of an inadequate margin of resection as evident by microscopic examination. Reoperation with a wider margin of resection resulted in no evidence of recurrence during 16 years of follow-up. In the other case involving the lip [14], the reason for the recurrence was explained by the absence of a fibrous capsule with tumor infiltration to the surrounding muscle. Reoperation was performed, and no recurrence was reported for 2 years thereafter. Preoperative diagnosis of intramuscular myxoma is difficult, and inadequate resection is associated with recurrence. Because complete resection of intramuscular myxoma is required, some surrounding soft tissue resection is advisable rather than injuring the fibrous capsule of the tumor.

In conclusion, this is the second reported case of an intramuscular myxoma of the tongue [13]. A correct preoperative diagnosis could not be obtained; however, typical findings of an intramuscular myxoma were seen on MRI. The clinical course of such lesions is not fully understood because of their rarity; therefore, long-term clinical follow-up is necessary.

## Figures and Tables

**Table 1:** Reported cases of intramuscular Myxoma in the head and neck.

No.	Author, year	Age/gender	Location	Preoperative examination	Size (cm)	Recurrence (postoperative months)	Follow-up period (months)
1	Enzinger, 1965 [3]	55/F	Sternocleidomastoid muscle	N/A	2.5 × 1.5 × 1.3	Absent	48
2	Rosin, 1973 [4]	44/M	Geniohyoid muscle	N/A	2.0	Absent	18
3	Kindblom et al., 1974 [5]	42/F	Occipitofrontalis muscle	N/A	N/A	Absent	144
4	Canalis et al., 1976 [6]	46/F	Lateral part of neck	N/A	N/A	Absent	24
5	Feldman, 1979 [7]	62/F	Posterior neck	N/A	3.0	Absent	120
6	Bedrosian et al., 1984 [8]	43/F	Masseter muscle	N/A	1.0	Absent	N/A
7	Wood, 1985 [9]	51/M	Posterior neck	N/A	N/A	Present (2)	192
8	Nisijima et al., 1985 [10]	16/F	Digastric muscle	US, CT	1.5, 1.5	Absent	120
9	Hashimoto et al., 1986 [11]	43/F	Massester muscle	N/A	2.0 × 1.0	Absent	55.2
10	Shugar et al., 1987 [12]	68/F	Levator scapulae muscle	CT, FNAC	4.0	Absent	12
11	Mockli et al., 1993 [13]	60/F	Tongue	FNAC	2.0	Absent	N/A
12	Orlandi et al., 1995 [14]	46/M	Orbicularis muscle	X-ray	3.0	Present (5)	24
13	Serrat et al., 1998 [15]	62/M	Temporalis muscle	CT, FNAC	5.0 × 4.0	Absent	9
14	van Roggen et al., 2001 [16]	56/M	Cheek	N/A	3.0	Absent	49
15	Crankson et al., 2002 [17]	5/F	Posterior triangle	US, CT	4.0	Absent	12
16	Robin et al., 2004 [18]	43/F	Temporalis muscle	CT, FNAC	3.5 × 2.6 × 2.0	Absent	18
17	Ozawa et al., 2004 [19]	22/M	Scalene muscle	US, CT	7.0 × 4.0 × 3.0	Absent	48
18	Ishoo, 2007 [20]	2/F	Posterior cervical triangle	CT, MRI, FNAC	5.0 × 4.0	Absent	24
19	Carlson et al., 2009 [21]	15/M	Stapedius muscle	MRI	0.6 × 0.4	N/A	N/A
20	Falavigna et al., 2009 [22]	64/F	Paraspinal muscle	CT, MRI	15.0	Absent	12
21	Papadogeorgakis, 2009 [23]	74/M	Masseter muscle	CT, FNAC	3.0 × 2.0	Absent	26
22	Patsiaoura et al., 2010 [24]	52/M	Mimetic muscle	MRI	2.0 × 1.3	Absent	8
23	Li et al., 2012 [25]	45/F	Posterior neck	US,CT	4.1 × 2.8 × 4.9	Absent	12
24	Kalshi et al., 2013 [26]	70/F	Sternocleidmastoid muscle	CT, MRI, FNAC	2.0 × 1.2 × 1.6	Absent	60
25	Higashida, 2014 [27]	51/M	Temporalis muscle	CT, MRI	6.5 × 4.0 × 3.0	Absent	6
26	Li et al., 2014 [28]	74/M	Hyoglossus muscle	CT	7.9 × 7.9	Absent	36
27	Tataryn et al., 2015 [29]	57/F	Cervical paraspinal musclature	MRI	2.0	Absent	N/A
28	Egami et al., 2015 [30]	76/M	Frontalis muscle	US	2.0	Absent	12
29	Rachidi et al., 2016 [31]	45/M	Levator scapulae and scalane	US, CT, MRI	2.7 × 2.5 × 1.4	Absent	30
30	Custódio et al., 2020 [32]	60/F	Masseter muscle	MRI	N/A	Absent	N/A
31	Nishi et al., 2021 [33]	59/M	Medial pterygoid muscle	MRI	1.75 × 1.36 × 0.71	Absent	6
32	Mijalis et al., 2021 [34]	86/F	Sternocleidmastoid muscle	CT, MRI, FNAC	3.5 × 3.4 × 4.8	Absent	0.25
33	Present case	23/F	Tongue	MRI	0.8 × 0.6	Absent	36

N/A: not applicable, CT: computed tomography, MRI: magnetic resonance imaging, FNAC: fine needle aspiration cytology.

## Data Availability

The data sets used during the current study are available from the corresponding author on reasonable request.
